# Numerical Analysis of Modeling Based on Improved Elman Neural Network

**DOI:** 10.1155/2014/271593

**Published:** 2014-06-18

**Authors:** Shao Jie, Wang Li, Zhao WeiSong, Zhong YaQin, Reza Malekian

**Affiliations:** ^1^Key Laboratory of Radar Imaging and Microwave Photonics, Ministry of Education, College of Electronic and Information Engineering, Nanjing University of Aeronautics and Astronautics, Nanjing 210016, China; ^2^Key Laboratory of Underwater Acoustic Signal Processing, Ministry of Education, Southeast University, Nanjing 210096, China; ^3^Department of Electrical, Electronic and Computer Engineering, University of Pretoria, Pretoria 0002, South Africa

## Abstract

A modeling based on the improved Elman neural network (IENN) is proposed to analyze the nonlinear circuits with the memory effect. The hidden layer neurons are activated by a group of Chebyshev orthogonal basis functions instead of sigmoid functions in this model. The error curves of the sum of squared error (SSE) varying with the number of hidden neurons and the iteration step are studied to determine the number of the hidden layer neurons. Simulation results of the half-bridge class-D power amplifier (CDPA) with two-tone signal and broadband signals as input have shown that the proposed behavioral modeling can reconstruct the system of CDPAs accurately and depict the memory effect of CDPAs well. Compared with Volterra-Laguerre (VL) model, Chebyshev neural network (CNN) model, and basic Elman neural network (BENN) model, the proposed model has better performance.

## 1. Introduction

The networks, communications, and television systems have entered the digital age. The class-D power amplifiers (CDPAs) [1] have become increasingly popular for audio applications because of their high power efficiency. Since the output transistors of CDPAs operate in the ohmic and cut-off regions, there exists nonlinearity in the system. One of the nonlinear phenomena is the intermodulation distortion (IMD) [2]. The CDPAs also have the memory effects, resulting from the node voltage and current depending on not only the current input but also the historical signals due to the existence of parameters with dynamic distribution [3]. The existence of memory effects [4] is often identified by imbalances between the corresponding upper and lower distortion products, such as the same order of IMD.

Behavioral modeling [5, 6] of nonlinear circuits and systems has received much attention in recent years. In behavioral modeling, the nonlinear component is generally considered as a “black box,” which is completely characterized by external responses, that is, in terms of input and output signals, through the use of relatively simple mathematical expressions. Behavioral modeling techniques provide a convenient and efficient means to predict system-level performance without the computational complexity of full circuit simulation or physical level analysis of nonlinear systems, thereby significantly speeding up the analysis process. The existing PA's behavioral models are mainly based on Volterra series or its expanded and simplified forms [7, 8]. However, its large number of coefficients complicates its practical implementation, which makes the standard Volterra series only limited to “weak” nonlinear PAs.

Owing to the fact that the neural networks are provided with available solutions for nonlinear function approximation, system identification, exclusive or and encoder problems, the study of PA's behavioral models based on neural networks has already been developed in recent years [9–12]. In order to well study the nonlinear characteristics of CDPAs, a new behavioral model based on improved Elman neural network (IENN) is proposed in this paper. In IENN, a self-connection of context nodes is added in this model, which could make the neurons more sensitive to the history of input data. The Chebyshev orthogonal polynomials [13, 14] instead of sigmoid functions are employed as the activation functions of hidden layer neurons to improve accuracy and the convergence rate of IENN, and the structure of IENN is simpler. The gradient descent (GD) algorithm is used to train the neural network model. Simulation results with two-tone signal, linear frequency modulated (LFM) signal, and binary phase shift keying (2PSK) signal as inputs have shown that the proposed model IENN could well depicts the nonlinear distortions of PAs.

The remainder of this paper is organized as follows. The basic Elman neural network (BENN) is introduced in Section 2. In Section 3, the new behavioral model based on IENN and the training algorithm of IENN is presented in detail. Simulation results using two-tone signal and broadband signals as input are given in Section 4. The conclusion is shown in Section 5.

## 2. The Basic Elman Neural Network

**Figure 1:**
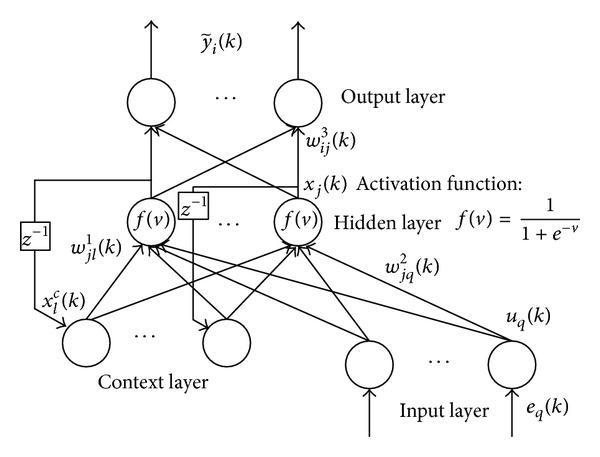
The architecture of BENN.

The architecture of BENN [15, 16] is illustrated in Figure 1, which is generally divided into four layers: input layer, hidden layer, context layer, and output layer. The feedforward loop consists of input layer, hidden layer, and output layer in which the weights connecting two neighboring layers are variable. There exists a back-forward loop between context layer and hidden layer, which makes the neural networks sensitive to the history of input data. In BENN, the context neurons can be treated as the memory units, so the model can manifest the memory effect of nonlinear system theoretically. Furthermore, because the dynamic characteristics of BENN are provided only by internal connections, there is no need to use the state as input or training signal, which makes BENN prior to static feedforward network.

## 3. The Behavioral Model Based on IENN

### 3.1. The Architecture of Improved Elman Neural Network

**Figure 2:**
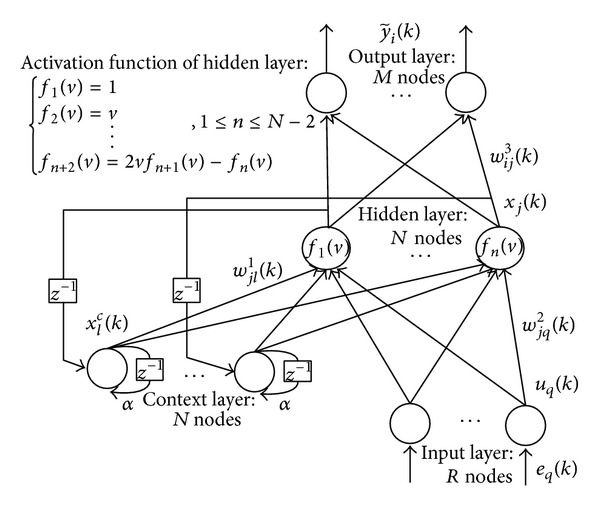
The architecture of IENN.

The architecture of IENN is presented in Figure 2; it is similar to BENN. To improve the learning speed and output accuracy, some changes are made. To better deliver the memory effect of the nonlinear system, a self-feedback is added to the context layer neurons with a feedback coefficient gain. This operation increases the memory depth and makes the model's output more sensitive to the history inputs. The value of Chebyshev orthogonal basis functions can easily be calculated by recursion operation, which is simpler than the sigmoid function. And the Chebyshev orthogonal basis functions have been used as active functions in many neural networks [14, 17, 18] for different applications and proved to be fast and accurate. We consider using the first category Chebyshev orthogonal basis as the activation function in the hidden layer instead of the sigmoid function in BENN. The research results have proved that the IENN model can simplify the computing complexity, reduce the training time, and enhance the convergence precision.

In IENN, the input layer has *R* nodes, the hidden layer and the context layer own *N* nodes, and the output layer possesses *M* nodes. The basic functions in each layer are as follows.

#### 3.1.1. Input Layer

In the input layer,

(1) $$\begin{array}{c} u_{q}\left(k\right)=e_{q}\left(k\right),\;q=1,2,\ldots ,R, \end{array}$$

where *k* represents the *k*th iteration step and *e*_*q*_(*k*) and *u*_*q*_(*k*) denote the input and the output of the input layer, respectively.

#### 3.1.2. Hidden Layer

The input of the *j*th hidden layer neuron is

(2) $$\begin{array}{c} v_{j}\left(k\right)=\overset{N}{\underset{l=1}{\sum }}w_{jl}^{1}{\left(k\right)x}_{l}^{c}\left(k\right)+\overset{R}{\underset{q=1}{\sum }}w_{jq}^{2}\left(k\right)u_{q}\left(k\right),\;j=1,2,\ldots ,N, \end{array}$$

where *x*_*l*_^*c*^(*k*) is the output of the *l*th context layer neuron, *w*_*jl*_^1^(*k*) represents the weight from the *l*th context layer neuron to the *j*th hidden layer neuron, and *w*_*jq*_^2^(*k*) represents the weight from the *q*th input layer neuron to the *j*th hidden layer neuron.

Since the input of Chebyshev orthogonal basis functions is defined within the interval [−1,1], the input of the hidden layer needs to be normalized. The normalization of *v*_*j*_(*k*) is defined as

(3) $$\begin{array}{c} {\overset{-}{v}}_{j}\left(k\right)=\frac{v_{j}\left(k\right)}{\max_{1\leq p\leq N}\left(\left|v_{p}\left(k\right)\right|\right)},\;j=1,2,\ldots ,N. \end{array}$$

The output of the *j*th hidden layer neuron is

(4) $$\begin{array}{c} x_{j}\left(k\right)=f_{j}\left[{\overset{-}{v}}_{j}\left(k\right)\right],\;j=1,2,\ldots ,N. \end{array}$$

The function *f*_*j*_(·) indicates the first category Chebyshev orthogonal basis functions given in Figure 2.

#### 3.1.3. Context Layer

In the context layer, the output is represented as

(5) $$\begin{array}{c} x_{l}^{c}\left(k\right)=\alpha x_{l}^{c}\left(k-1\right)+x_{l}\left(k-1\right),\;l=1,2,\ldots ,N, \end{array}$$

where 0 ≤ *α* ≤ 1 is the self-connection feedback gain of the context layer. When *α* = 0, this network is reductive into the BENN.

#### 3.1.4. Output Layer

The output ${\tilde{y}}_{i}(k)$ of IENN can be expressed as

(6) $$\begin{array}{c} {\tilde{y}}_{i}\left(k\right)=\overset{N}{\underset{j=1}{\sum }}w_{ij}^{3}\left(k\right)x_{j}\left(k\right),\;i=1,2,\ldots ,M, \end{array}$$

where *w*_*ij*_^3^(*k*) denotes the weight from *j*th hidden layer neuron to *i*th output layer neuron.

### 3.2. Training Algorithm

Training of the neural networks has been developed rapidly in recent years [19–21]. The gradient descent (GD) algorithm, as a basic approach for training neural networks in many areas, searches the parameter space of the network in the steepest descent way to minimize the error between the network output and the desired output [22]. In IENN, the gradient descent algorithm is used to update the weights. Assume that the actual system output vector is **y**(*k*) = [*y*_1_(*k*),*y*_2_(*k*),…,*y*_*M*_(*k*)]^*T*^ (*T* is used for transpose) and the *k*th iteration of IENN model output vector is $\tilde{y}\left(k\right)={\left[{\tilde{y}}_{1}\left(k\right),{\tilde{y}}_{2}\left(k\right),\ldots ,{\tilde{y}}_{M}\left(k\right)\right]}^{T}$. The error-function, namely, the sum of squared error (SSE), is defined as

(7) $$\begin{array}{c} \text{SSE}\left(k\right)=\frac{1}{2}{\left[y\left(k\right)-\tilde{y}\left(k\right)\right]}^{T}\left[y\left(k\right)-\tilde{y}\left(k\right)\right]. \end{array}$$

By using the partial derivative of error-function with respect to the weight parameters, the increments of the weights are as follows:

(8) Δwjl1(k)=η1∑i=1M[δi(k)wij3(k)]∂xj(k)∂wjl1(k),Δwjq2(k)=η2λj(k)uq(k),Δwij3(k)=η3δi(k)xj(k),

with

(9) $$\begin{array}{c} \delta_{i}\left(k\right)=y_{i}\left(k\right)-{\tilde{y}}_{i}\left(k\right),\\ \lambda_{j}\left(k\right)=\overset{M}{\underset{i=1}{\sum }}\left[\delta_{i}\left(k\right)w_{ij}^{3}\left(k\right)\right]\frac{f_{j}^{\prime }\left(\cdot \right)}{\max_{1\leq p\leq N}\left(\left|v_{p}\left(k\right)\right|\right)},\\ \frac{\partial x_{j}\left(k\right)}{\partial w_{jl}^{1}\left(k\right)}=\alpha \frac{\partial x_{j}\left(k-1\right)}{\partial w_{jl}^{1}\left(k-1\right)}+\frac{f_{j}^{\prime }\left(\cdot \right)x_{l}\left(k-1\right)}{\max_{1\leq p\leq N}\left(\left|v_{p}\left(k\right)\right|\right)}, \end{array}$$

where *f*_*j*_′(·) is the first derivative of the normalized input of hidden layer neurons ${\overset{-}{v}}_{j}(k)$. *η*_1_, *η*_2_, and *η*_3_ represent the learning rate of *w*_*jl*_^1^, *w*_*jq*_^2^, and *w*_*ij*_^3^, respectively.

In order to well analyze the error of system output and IENN output, the transient absolute error vector **σ**(*k*) is defined as

(10) $$\begin{array}{c} \sigma \left(k\right)=\left|y\left(k\right)-\tilde{y}\left(k\right)\right|. \end{array}$$

The mean error of **σ**(*k*) is

(11) $$\begin{array}{c} \overset{-}{\sigma }\left(k\right)=\frac{\left[\sum_{i=1}^{M}\left|y_{i}\left(k\right)-{\tilde{y}}_{i}\left(k\right)\right|\right]}{M}. \end{array}$$

### 3.3. Training Steps of IENN

By using the GD method, the training steps to determine the optimal number of neurons in hidden layer are as follows. The initial values of *α*, *η*_1_, *η*_2_, and *η*_3_ are got by continuous testing.

**Step 1 .**

Prepare the training input and output data. Set the initial number for neurons *N* = 4 in hidden layer, define the maximum neurons *N*_max⁡_ = 100 in hidden layer, and the maximum iteration step *K*_max⁡_ = 100. The threshold value of SSE is *ε*_min⁡_.

**Step 2 .**

Set the self-connection feedback coefficient *α* = 0.1; weights of IENN *w*_*jl*_^1^(1), *w*_*jq*_^2^(1), and *w*_*ij*_^3^(1) as constant 0 and their learning rate *η*_1_ = *η*_2_ = *η*_3_ = 0.01; the partial derivative ∂*x*_*j*_(0)/∂*w*_*jl*_^1^(0) = 0. The initial iteration step *k* = 0.

**Step 3 .**

Increase the number of iteration step *k* = *k* + 1; if *k* > *K*_max⁡_, end the training process. According to formulas (1) to (7), calculate the value of every neuron in every layer and the SSE(*k*) of *k*th iteration step. If the SSE(*k*) is less than *ε*_min⁡_, end the training process.

**Step 4 .**

Calculate the increments of the weights shown in formulas (8); then the weights are updated as *w*_*jl*_^1^(*k* + 1) = *w*_*jl*_^1^(*k*) + Δ*w*_*jl*_^1^(*k*), *w*_*jq*_^2^(*k* + 1) = *w*_*jq*_^2^(*k*) + Δ*w*_*jq*_^2^(*k*), and *w*_*ij*_^3^(*k* + 1) = *w*_*ij*_^3^(*k*) + Δ*w*_*ij*_^3^(*k*). Jump to Step 3.

**Step 5 .**

Increase the number of neurons *N* in hidden layer; if *N* > *N*_max⁡_, end the training process. Jump to Step 2.

## 4. Simulation Results and Analysis

**Figure 3:**
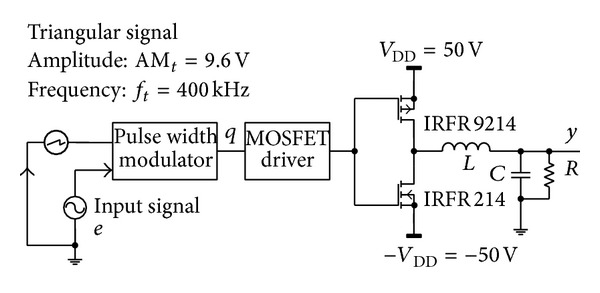
The circuit of half-bridge CDPA.

In order to verify the correctness and reliability of IENN model, the training sample sequences are achieved from input *e* of half-bridge CDPA shown in Figure 3.

As shown in Figure 3, the PWM signal *q* is produced by the comparison between the two-tone signal and the triangular signal. The frequency and amplitude of triangular signal are *f*_*t*_ = 400 kHz and AM_*t*_ = 9.6 V. The output signal of CDPA is termed as **y**. A group of the training data is extracted by the sampling frequency *f*_*s*_ = 1 MHz. The testing data has the same length and sampling frequency with the training data; the difference is the starting time. In the simulation results, the testing data's starting time is treated as 0 ms.

### 4.1. Optimal Neurons Number in Hidden Layer

**Figure 4:**
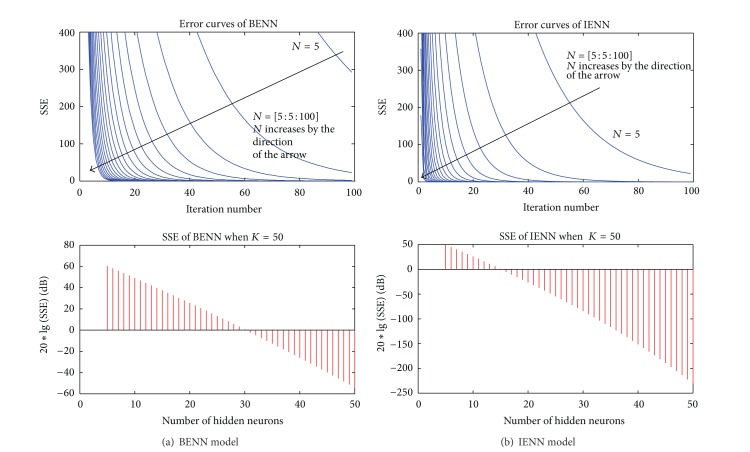
SSE varying with iteration number *K* and number of hidden neurons *N*.

In order to determine the optimal neurons number in hidden layer of two models, by using the training data of two-tone signal, with the frequencies of the two-tone signal being *f*_1_ = 4.36 kHz and*f*_2_ = 30 kHz, their amplitudes being AM_1_ = AM_2_ = 4 V, and the signal length being 0.5 ms, the relationship between SSE(*k*) and the number of hidden layer neurons is studied. When the maximum iteration step *K*_max⁡_ = 100, the number of hidden neurons *N* increases from 5 to 100 with the interval of 5; do not set the error threshold of SSE(*k*); the error curves of SSE(*k*) are shown in the left side of Figure 4. The lower side of Figures 4(a) and 4(b) shows the SSE(*k*) changing with hidden neurons number; when the iteration step *K* = 50, the neurons number in hidden layer increases from 5 to 50 with the interval of 1.

It can be seen in the top of Figures 4(a) and 4(b) that, with the increase of the iteration step, the error curves of SSE(*k*) drop rapidly. The larger the number of hidden neurons *N* is, the faster SSE(*k*) decreases, and the less iteration steps needed to reach the same SSE are. The comparison between BENN and IENN shows that IENN has faster convergence rate than BENN. For example, to reach the same SSE(*k*) of 50, with the same number of hidden neurons *N* = 15, BENN needs an iteration number of about 55, while IENN needs an iteration number of only 25; the calculation of IENN is reduced to almost half of BENN.

On the lower side of Figures 4(a) and 4(b), when BENN and IENN have the same number of iteration; to achieve the same SSE(*k*), IENN needs less neurons in hidden layer than BENN. For instance, to reach the logarithmic SSE(*k*) of 0 dB, BENN needs 30 neurons in hidden layer while IENN needs only 15 neurons in hidden layer.

In consideration of the convergence rate and the calculation, *N* = 25 is chosen as the number of hidden layer neurons in the following discussion.

### 4.2. Simulation Analysis of Four Models with Two-Tone Signal Input

**Figure 5:**
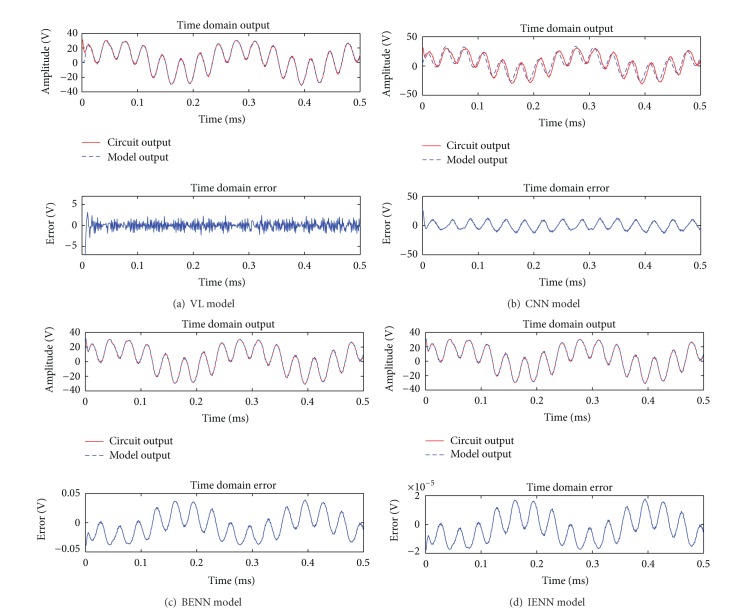
Comparison among four behavioral models in time domain with two-tone signal input.

The Volterra-Laguerre (VL) model [7] and the Chebyshev neural network (CNN) model [17, 23, 24] are introduced to be compared with the BENN and IENN model. The VL model is proposed in [7]. There are two parameters in this model: the number of Laguerre orthogonal functions *K* and the pole of Laguerre functions *λ* (|*λ*| < 1). When *K* = 3, this model cannot reconstruct the output well. Here, we choose *K* = 5 and *λ* = 0.97; there are 605 parameters needed to be estimated. The CNN model in [23] employs a group of Chebyshev orthogonal polynomials to activate the hidden layer neurons, and based on the GD method, the iterative training formula is obtained. For three neural network models, set the number of hidden layer neurons *N* = 25 and the iteration step *K*_max⁡_ = 50. Using the two-tone signal as input, the simulation results of four behavioral models in time domain are shown in Figure 5.

In Figure 5, the time domain error is the transient error $y-\tilde{y}$. Values of the mean error $\overset{-}{\sigma }$ and the maximum transient error *σ*_max⁡_ of four models are listed in Table 1 with two-tone signal input.

It can be seen in Figure 5 and Table 1 that IENN is the most accurate model among four models. The VL and CNN model cannot reconstruct the output signal accurately, and the transient error is very large at the beginning of the data. Both the BENN and IENN have stable approximation capability; under the same conditions, IENN is more precise than BENN. The final maximum transient error of BENN is 0.0391 V, while it is only 1.78 × 10^−5^ V in IENN.

**Figure 6:**
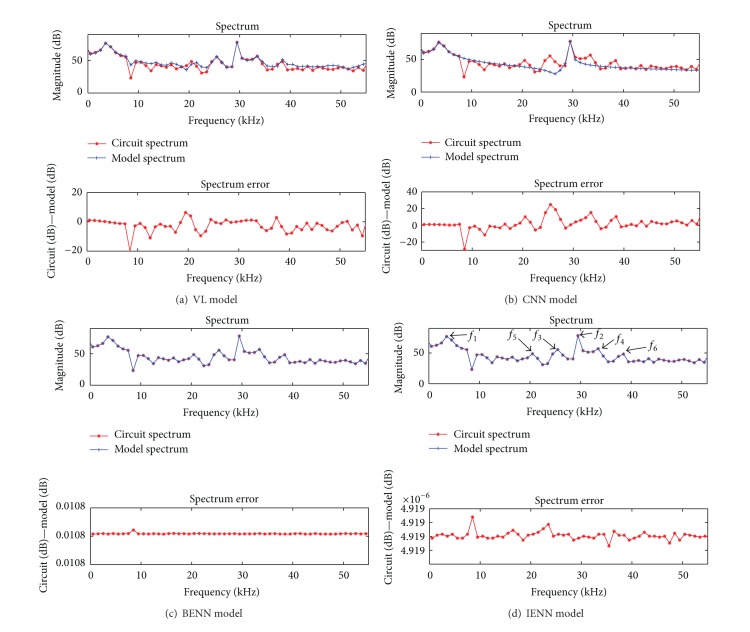
Comparison among four behavioral models in frequency domain with two-tone signal input.

The two-tone signal is often used to study the memory effect of the nonlinear system [4, 25] since the IMD of the signal is easy to measure. When a two-tone signal is used as training data, the simulation results of four models in frequency domain are given in Figure 6.

In Figure 6(d), *f*_1_ = 4.36 kHz and *f*_2_ = 30 kHz are the input two-tone signal's frequencies. *f*_3_ = *f*_2_ − *f*_1_ and *f*_4_ = *f*_2_ + *f*_1_ are the second order IMD (IMD2). *f*_5_ = *f*_2_ − 2*f*_1_ and *f*_6_ = *f*_2_ + 2*f*_1_ are the third order IMD (IMD3). The existence of IMD means the system is nonlinear and the asymmetry of IMD demonstrates the memory effect of the system. The circuit output spectrum and the spectrum error are listed in Table 2.

As shown in Figure 6 and Table 2, the spectrum error at IMD2 and IMD3 of VL model is a little large, the asymmetry between the upper and lower sidebands has been weakened, and some of the memory effect characteristics are lost. The short memory length of VL model is the reason for this. But the number of parameters in this model is already large; if the memory length increases, the parameters will increase rapidly. The spectrum of CNN in Figure 6(b) shows that it has lost almost all the information of the IMD. Since the CNN model is a feedforward neural network, the output of the model is only related to the input at present moment; it cannot express the previous influence of the inputs on the output, namely, that the CNN model cannot demonstrate the memory effect. The spectrum errors of BENN and IENN model are stable; under the same conditions, the spectrum error of BENN is 0.011 dB, and IENN is 4.92 × 10^−6^ dB. The IENN is much more accurate than BENN.

### 4.3. Simulation Analysis of Four Models with LFM Signal Input

**Figure 7:**
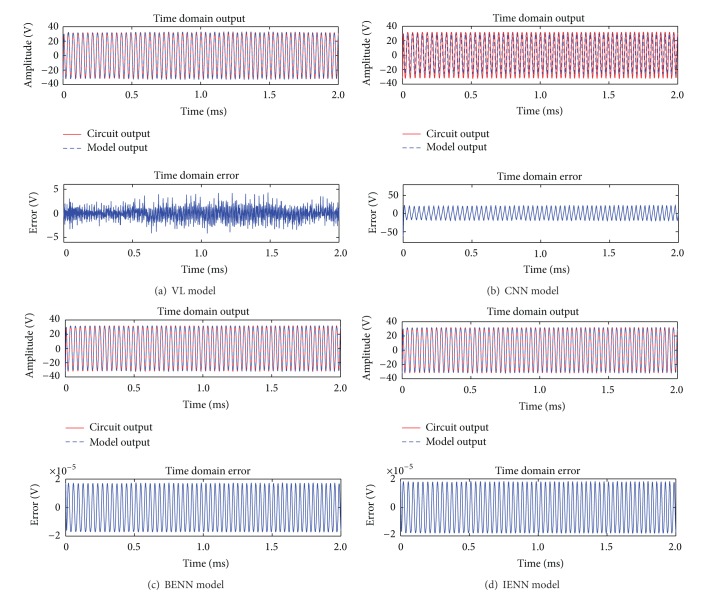
Comparison among four behavioral models in time domain with LFM signal input.

**Figure 8:**
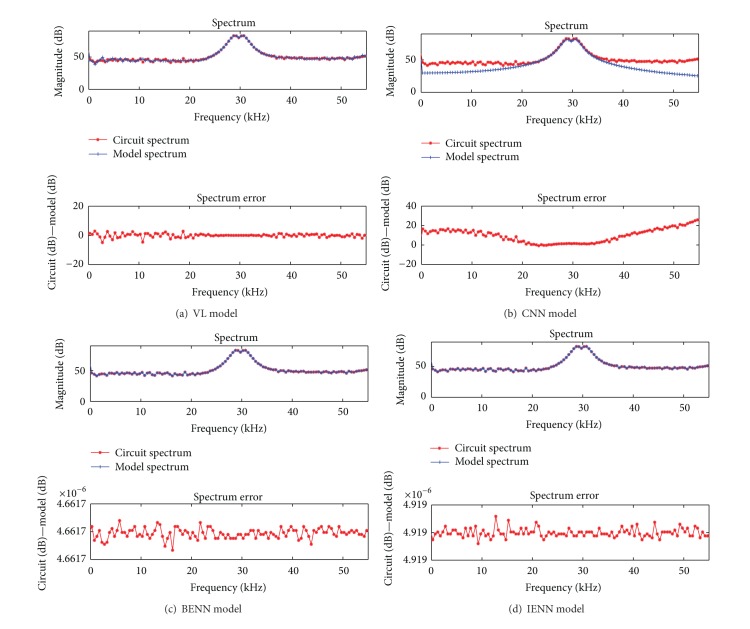
Comparison among four behavioral models in frequency domain with LFM signal input.

For the experimental validation, the LFM signal is used as input *e* of half-bridge CDPA, whose center frequency is 30 kHz, amplitude is 8.5 V, bandwidth is 4 kHz, and training data length is 2.0 ms. Other parameters of the simulation are the same as above. Using the LFM signal as training samples, the simulation results of four behavioral models in time domain are shown in Figure 7; the results in frequency domain are given in Figure 8. The mean error $\overset{-}{\sigma }$ and the maximum transient error *σ*_max⁡_ of four models in time domain are listed in Table 3, and the average spectrum errors and the maximum spectrum errors are listed in Table 4.

As shown in Figure 7 and Table 3, when the LFM signal is used as input of CDPA, the transient error of the VL and CNN model is very huge, and the output signal cannot be reconstructed accurately. In the same conditions of the number of hidden neurons *N* = 25 and the iteration step *K*_max⁡_ = 50, the time domain errors of both IENN and BENN model are basically the same and the final maximum transient error of BENN is 1.74 × 10^−5^ V, while it is 1.83 × 10^−5^ V in IENN. Both the BENN and IENN have stable approximation capability.

As shown in Figure 8 and Table 4, using the training data of LFM signal, the spectrum error of the VL and CNN model is similarly very large and has lost the correct information of the memory effect in frequency domain. The spectrum errors of BENN and IENN model are stable; under the same simulation conditions, the maximum spectrum error of BENN is 4.66 × 10^−6^ dB, and IENN is 4.92 × 10^−6^ dB. The performance of IENN and BENN model in frequency domain is almost the same.

### 4.4. Simulation Analysis of Four Models with 2PSK Signal Input

**Figure 9:**
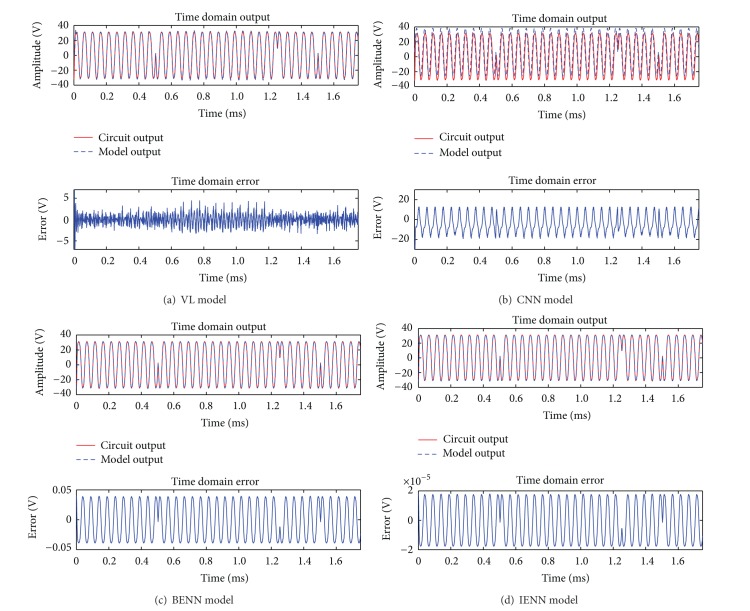
Comparison among four behavioral models in time domain with 2PSK signal input.

**Figure 10:**
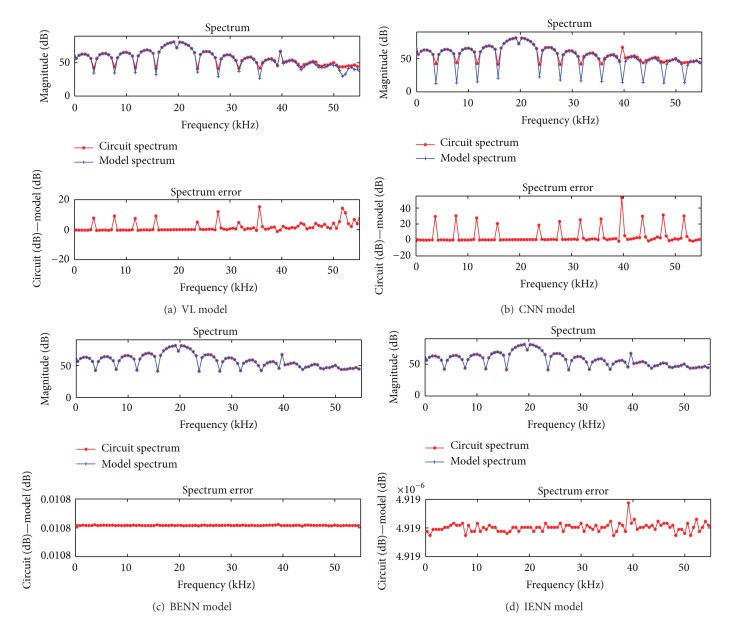
Comparison among four behavioral models in frequency domain with 2PSK signal input.

In further experiments, a 2PSK signal is used as input *e* of half-bridge CDPA, whose carrier frequency is 20 kHz, amplitude is 8.5 V, digital baseband signal is a 7-bit pseudorandom sequence (*m* sequence), baseband symbol width is 0.25 ms, and testing data length is 1.75 ms. Other parameters of the models are the same as above too. Using the 2PSK signal input, the simulation results of four behavioral models in time domain are shown in Figure 9, and the results in frequency domain are given in Figure 10. The mean error $\overset{-}{\sigma }$ and the maximum transient error *σ*_max⁡_ of four models in time domain are listed in Table 5, and the average spectrum error and the maximum spectrum error are listed in Table 6.

It can be seen in Figure 9 and Table 5 that with a 2PSK signal input, the VL and CNN model cannot reconstruct the CDPA output accurately, and the transient error is still very large. Under the same conditions that the number of hidden neurons is 25 and the iteration step is 50, IENN model is more precise than BENN model. The final maximum transient error of BENN is 0.0398 V, while it is only 1.81 × 10^−5^ V in IENN. IENN model is the most accurate model among four models.

As shown in Figure 10 and Table 6, using the training samples of 2PSK signal, the spectrum errors of the VL and CNN model are still very large, and the memory effect of CDPA cannot be demonstrated by these models. The spectrum errors of BENN and IENN model are steady. Under the same conditions, the maximum spectrum error of BENN is 0.011 dB, and IENN is 4.92 × 10^−6^ dB. At this point, the IENN model is much more accurate than BENN model.

The comparison among four behavioral models under the condition of different input signals and the same simulation parameters shows that the proposed IENN model is the most accurate model for analyzing the nonlinearity and memory effect of the CDPAs in both time domain and frequency domain.

## 5. Conclusions

In this paper, a behavioral modeling based on IENN is proposed to describe the nonlinearity and memory effect of CDPAs. In IENN, a group of Chebyshev orthogonal basis functions is employed to activate hidden layer neurons to improve the learning speed and the accuracy and also to simplify the structure of model. A self-connection of context nodes is added to make the output more sensitive to the history of input data.

According to the simulation results, it can be seen that, to reach the same error threshold, compared to BENN, IENN needs fewer hidden layer neurons and less iteration steps. It means that IENN has fast learning speed and can use simpler network structure to achieve the same requirements than many other neural networks. Using the same number of hidden layer neurons and iteration number, simulation results by using the training data of two-tone, LFM and 2PSK signal have shown that the IENN is superior to VL, CNN, and BENN model in accuracy; it has reconstructed the nonlinear CDPA system with almost no transient or spectrum error; the memory effect is also visualized. Above all, the proposed IENN model is an effective, efficient, and simple behavioral model for nonlinear systems.

## Figures and Tables

**Table 1:** Mean error $\overset{-}{\sigma }$ and maximum transient error *σ*_max⁡_ of four behavioral models with two-tone signal input.

Model	$\overset{-}{\sigma }$ (V)	*σ*_max⁡_ (V)	Condition
VL	0.9272	31.3400	*K* = 5, *λ* = 0.97

CNN	5.9397	27.6531	*N* = 25, *K*_max⁡_ = 50
BENN	0.0182	0.0391	
IENN	8.2434*e* − 06	1.7754*e* − 05	

**Table 2:** Spectrum of the circuit and spectrum error of four behavioral models with two-tone signal input.

Freq. (Hz)	Circuit spectrum (dB)	VL (dB)	CNN (dB)	BENN (dB)	IENN (dB)
*f*_1_	71.83	−0.22	0.62	0.0108	4.919*e* − 06
*f*_2_	65.15	0.24	2.39		
*f*_3_	54.07	−0.58	23.56		
*f*_4_	46.33	−3.19	5.55		
*f*_5_	42.58	−3.57	5.06		
*f*_6_	45.01	−4.56	7.17		

**Table 3:** Mean error $\overset{-}{\sigma }$ and maximum transient error *σ*_max⁡_ of four behavioral models with LFM signal input.

Model	$\overset{-}{\sigma }$ (V)	*σ*_max⁡_ (V)	Condition
VL	0.9564	4.2593	*K* = 5, *λ* = 0.97

CNN	11.0047	61.6258	*N* = 25, *K*_max⁡_ = 50
BENN	1.1539*e* − 05	1.7378*e* − 05	
IENN	1.2176*e* − 05	1.8337*e* − 05	

**Table 4:** Spectrum error of four behavioral models with LFM signal input.

Model	Average error (dB)	Max. error (dB)	Condition
VL	2.3714	24.2021	*K* = 5, *λ* = 0.97

CNN	33.0427	77.3836	*N* = 25, *K*_max⁡_ = 50
BENN	4.6617*e* − 06	4.6617*e* − 06	
IENN	4.9190*e* − 06	4.9190*e* − 06	

**Table 5:** Mean error $\overset{-}{\sigma }$ and maximum transient error *σ*_max⁡_ of four behavioral models with 2PSK signal input.

Model	$\overset{-}{\sigma }$ (V)	*σ*_max⁡_ (V)	Condition
VL	1.0395	31.9100	*K* = 5, *λ* = 0.97

CNN	8.6677	41.8680	*N* = 25, *K*_max⁡_ = 50
BENN	0.0271	0.0398	
IENN	1.2309*e* − 05	1.8071*e* − 05	

**Table 6:** Spectrum error of four behavioral models with 2PSK signal input.

Model	Average error (dB)	Max. error (dB)	Condition
VL	8.1023	33.1085	*K* = 5, *λ* = 0.97

CNN	14.5702	52.7683	*N* = 25, *K*_max⁡_ = 50
BENN	0.0108	0.0108	
IENN	4.9190*e* − 06	4.9190*e* − 06	
